# Normal reference values for thoracic and abdominal aorta and main pulmonary artery dimensions by cardiovascular magnetic resonance: the Framingham heart study

**DOI:** 10.1186/1532-429X-15-S1-P256

**Published:** 2013-01-30

**Authors:** Michael L Chuang, Philimon Gona, Carol J Salton, Connie W Tsao, Susan B Yeon, Christopher J O'Donnell, Warren J Manning

**Affiliations:** 1 NHLBI's Framingham Heart Study, Framingham, MA, USA; 2 Cardiovascular Division, Beth Israel Deaconess Medical Center, Boston, MA, USA

## Background

Enlargement of the aorta or main pulmonary artery( MPA) is associated with cardiopulmonary disease, and an increased MPA-to-ascending aorta ratio is associated with pulmonary hypertension. We sought to determine mean and upper 90th percentile (p90) diameters of the thoracic and abdominal aorta and MPA in a longitudinally-followed adult cohort without clinical cardiopulmonary disease.

## Methods

1794 Framingham Heart Study Offspring cohort members (65±9 yrs, 844 men) underwent ECG-gated, free breathing T2-weighted black-blood TSE at 1.5T (Philips Gyroscan NT, TR=3RR, TE=45ms, trigger delay=75ms (thorax) or 125ms (abdomen), 1.03x0.64-mm^2^ in-plane resolution, THK=5mm, Gap=5 (abdomen) or 10mm (thorax)). Ascending (ASC) and descending thoracic (DTA) aortic and MPA diameters were measured at MPA-bifurcation level, abdominal (ABD) aorta was measured 5 mm above renal artery origins. We determined sex-specific mean, SD and p90 values for vessel diameters and MPA/ASC ratio in a healthy referent group free of hypertension (SBP≥140 or DBP≥90 mmHg or on medication), obesity (body mass index≥30 kg/m^2^), emphysema, prevalent myocardial infarction or heart failure, and any smoking history. Men were compared to women using 2-sample t test. We also indexed vessel diameters to sex-and-vessel specific allometric powers of height (HT^β^); β's were determined by linear regression of log(HT) to log(diameter). Indexation to HT^β^ was selected since indexation to HT or body surface area (BSA) resulted in significant inverse correlations of vessel diameters to HT and/or BSA.

## Results

370 Offspring (62±9 yrs) met referent-group criteria. Men had greater aortic (at all levels) and MPA diameters than women both before and after indexation to HT^β^. The β values corresponding to ASC, DTA, ABD and MPA were 0.22, 0.30, 0.11 and 0.29, respectively, in men, and 0.10, 0.39, 0.37 and 0.48 in women. Vessel diameters indexed to HT^β^ were correlated with neither HT nor BSA. The MPA/ASC ratio did not differ between sexes. The mean±SD and upper limits (p90) for raw and indexed vessel diameters and MPA/ASC ratio are shown (Table).

**Table 1:** 

	Men	p90 (Men)	Women	p90 (Women)
ASC, mm	31.1±2.9	34.9	28.5±3.1	31.8
DTA, mm	23.0±2.0	25.3	20.2±1.8	22.7
ABD, mm	17.9±1.6	20.0	15.3±1.5	17.3
MPA, mm	23.4±2.9	26.6	21.3±3.1	24.2
ASC/HT^β^	27.5±2.6	30.9	27.1±2.9	30.4
DTA/ HT^β^	19.4±1.7	21.4	16.7±1.5	18.6
ABD/ HT^β^	16.8±1.5	18.9	12.8±1.3	14.4
MPA/ HT^β^	19.9±2.5	22.5	16.9±2.4	19.1
MPA/ASC	0.76±0.10	0.88	0.75±0.15	0.87

## Conclusions

We present CMR-derived sex-specific upper 90th percentile values for aortic and MPA diameters and MPA/ASC ratio derived from a cohort of longitudinally-followed, community dwelling adults free of clinical cardiac and pulmonary disease. These p90 thresholds may be useful for identification of cardiopulmonary pathology.

## Funding

Supported in part by grant RO1 AG17509 and by subcontract N01-HC-38038 from the National Institutes of Health.

